# Book review on leadership and self deception

**DOI:** 10.1002/acm2.13247

**Published:** 2021-05-04

**Authors:** Justin Gagneur

**Affiliations:** ^1^ Radiation Oncology Mayo Clinic in Arizona Phoenix AZ USA

## 1 INTRODUCTION

Since the advent of the AAPM Medical Physics 3.0 Initiative, AAPM has been focused on how physicists can bring additional value to their respective organizations. This could be as simple as new approaches to QA procedures or as complex as expanding greatly beyond our traditional roles in the clinic. As recently highlighted in the Medical Physics Leadership Academy Journal Club, the Arbinger Institute teaches leadership, change management, and conflict resolution among other skills. This meshes well with the MPLA Journal Club mission of teaching Influencing, Conflict Management, Adaptability, Initiative, and Self Confidence. In their seminal book *Leadership and Self‐Deception,* they teach how to navigate these challenges through a unique narrative format. Other books (*Dare to Lead, Smarter Faster Better, Turn the Ship Around*) also help teach critical leadership skills, but *Leadership and Self‐Deception* lays an excellent foundation to be expanded on with further reading and exploration.

## 2 PURPOSE AND AUDIENCE

Through its narrative *Leadership and Self‐Deception* walks the reader through critical leadership, change management, and conflict resolution skills. It offers practical relatable examples and allows the reader opportunity to reflect on these skills. This book and the skills that it teaches are applicable to all medical physicists. With the impending alternative payment method, administrators are going to be looking for how highly paid physicists bring value to not only the department but also to the organization. Physicists have unique perspectives in the clinic. By learning and practicing these skills, physicists can step out of the traditional technical role and partner with administrators and other leaders. Many conflicts and change management issues within a department are born out of a lack of clarity amongst all parties. Uniquely Physicists speak everyone’s language and can be the bridge between groups to help them understand each other’s perspective. Using these skills, Physicists can actively manage interactions between groups and increase intradepartmental communication.

## 3 CONTENTS AND HIGHLIGHTS

*Leadership and Self‐Deception* follows the hypothetical journey of Tom Callum through his orientation and integration into the Zagrum Company. Over a short 170 pages, the reader follows Tom’s journey of self‐discovery. It starts simply with the realization that there is a problem, rapidly evolves into how to identify issues before they become real problems, and then how to effectively deal with them. The narrative format and many practical examples draw the reader in and elicits comparisons and introspection on situations that the reader has experienced. An example of this is developing the skill to realize when you are warping your perceptions to match a predefined narrative (i.e., self‐deception). How often have you looked at a treatment plan and decided that the dosimetrist did not “try hard enough”? Using the lessons from *Leadership and Self‐Deception* you will develop the skill to short circuit that thought process and partner with the dosimetrist to get to the root cause of the issue.

## 4 CRITICAL ASSESSMENT

In the modern radiation oncology department, Physicists more and more are expected to operate outside of their traditional realms. Administrators, Leaders, and Physicians look to us and ask what value do you bring to the organization? QA, clinical work, and implementing new technology are increasingly not enough to tilt the value equation in our favor. *Leadership and Self‐Deception* provides a framework and set of tools to not only make us more effective in our traditional roles but also to aid us in branching out as professionals. Using the skills outlined in the book we can be more than a technical resource. We can be a coequal in leadership and management meetings and lead the way for departmental change. As an example, I coordinate and lead all ARIA upgrades for our clinic. On the surface this appears to be a technical traditional physicist role, but through the lens and lessons of *Leadership and Self‐Deception* it is so much more. It is meetings with all departmental groups to listen to concerns, develop training plans, and interface with the vendor resources. Our role then evolves into a partnership with management and administration.

I highly recommend this book to every physicist, trainee, and student. The skills and narrative are applicable to all aspects of Medical Physics. By way of example, our department used *Leadership and Self‐Deception* to kick off a leadership book club for the management team. This included representation from Administration, Nursing, Dosimetry, Therapy, and Physics. The team read the book and once a month convened to discuss what they had learned. This open forum allowed the different leads to practice the skills that they had recently learned and to hear the trials and success that others had experienced. It also provided an opportunity to showcase a physicist’s unique perspective on the clinic. One of the leads brought up a scenario for how to deal with a therapist who was not following a technical procedure. As a physicist this was a situation that I was very comfortable with. Through discussion we walked through whether this was a competence problem or a clarity problem. Competence issues are straight forward to correct with further training. Often though a lack of clarity is why a procedure is not followed. If the therapist does not understand why the procedure is important why should not shortcuts be made? Physicists excel in explaining the technical details and the why. In that moment the lead therapist and I made a connection. We both realized that by tackling the problem together we could come to a better solution than if we worked on it separately. This is a prime example of how physicists can bring great value to an organization. Physicists often have unique and valuable perspectives and this type of forum allows us to showcase that.

